# Prevalence and Antimicrobial Susceptibility of *Vibrio parahaemolyticus* Isolated from Short Mackerels (*Rastrelliger brachysoma*) in Malaysia

**DOI:** 10.3389/fmicb.2017.01087

**Published:** 2017-06-13

**Authors:** Chia W. Tan, Tan T. H. Malcolm, Chee H. Kuan, Tze Y. Thung, Wei S. Chang, Yuet Y. Loo, Jayasekara M. K. J. K. Premarathne, Othman B. Ramzi, Mohd F. S. Norshafawatie, Nordin Yusralimuna, Yaya Rukayadi, Yoshitsugu Nakaguchi, Mitsuaki Nishibuchi, Son Radu

**Affiliations:** ^1^Department of Food Science, Faculty of Food Science and Technology, Universiti Putra MalaysiaSelangor, Malaysia; ^2^Department of Livestock and Avian Science, Faculty of Livestock, Fisheries and Nutrition, Wayamba University of Sri LankaMakandura, Sri Lanka; ^3^Center for Southeast Asian Studies, Kyoto UniversityKyoto, Japan; ^4^Laboratory of Food Safety and Food Integrity, Institute of Tropical Agriculture and Food Security, Universiti Putra MalaysiaSelangor, Malaysia

**Keywords:** *Vibrio parahaemolyticus*, finfish, MPN, antibiotic susceptibility, MAR

## Abstract

Numerous prevalence studies and outbreaks of *Vibrio parahaemolyticus* infection have been extensively reported in shellfish and crustaceans. Information on the quantitative detection of *V. parahaemolyticus* in finfish species is limited. In this study, short mackerels (*Rastrelliger brachysoma*) obtained from different retail marketplaces were monitored with the presence of total and pathogenic strains of *V. parahaemolyticus*. Out of 130 short mackerel samples, 116 (89.2%) were detected with the presence of total *V. parahaemolyticus* and microbial loads of total *V. parahaemolyticus* ranging from <3 to >10^5^ MPN/g. Prevalence of total *V. parahaemolyticus* was found highest in wet markets (95.2%) followed by minimarkets (89.1%) and hypermarkets (83.3%). Pathogenic *V. parahaemolyticus* strains (*tdh*+ and/or *trh*+) were detected in 16.2% (21 of 130) of short mackerel samples. The density of *tdh*+ *V. parahaemolyticus* strains were examined ranging from 3.6 to >10^5^ MPN/g and microbial loads of *V. parahaemolyticus* strains positive for both *tdh* and *trh* were found ranging from 300 to 740 MPN/g. On the other hand, antibiotic susceptibility profiles of *V. parahaemolyticus* strains isolated from short mackerels were determined through disc diffusion method in this study. Assessment of antimicrobial susceptibility profile of *V. parahaemolyticus* revealed majority of the isolates were highly susceptible to ampicillin sulbactam, meropenem, ceftazidime, and imipenem, but resistant to penicillin G and ampicillin. Two isolates (2.99%) exhibited the highest multiple antibiotic resistance (MAR) index value of 0.41 which shown resistance to 7 antibiotics. Results of the present study demonstrated that the occurrence of pathogenic *V. parahaemolyticus* strains in short mackerels and multidrug resistance of *V. parahaemolyticus* isolates could be a potential public health concerns to the consumer. Furthermore, prevalence data attained from the current study can be further used to develop a microbial risk assessment model to estimate health risks associated with the consumption of short mackerels contaminated with pathogenic *V. parahaemolyticus*.

## Introduction

*Vibrio parahaemolyticus* is found naturally in the marine environment and may accumulate in seafood especially shellfish at high concentrations. Consumption of foods contaminated with high concentration of total *V. parahaemolyticus* and/or pathogenic *V. parahaemolyticus* can cause gastrointestinal infections. An open wound in skin comes in contact with *V. parahaemolyticus* is suggested as an infection pathway as well. Major syndromes caused by *V. parahaemolyticus* include gastroenteritis, wound infection, and septicaemia. Typical symptoms of the gastroenteritis may include abdominal pain, diarrhea, nausea, and fever. Based on the Foodborne Diseases Active Surveillance Network (FoodNet) data and Morbidity and Mortality Weekly Report (MMWR) published by Centers for Disease Control and Prevention (CDC) in the United States during the year 2016, *V. parahaemolyticus* is the major foodborne pathogen compared to other *Vibrio* isolates and it is estimated *V. parahaemolyticus* causes about 34,664 episodes of domestically acquired foodborne illness annually in the United States (Scallan et al., 2011; Huang et al., 2016).

In term of pathogenicity, thermostable direct hemolysin (TDH) and thermostable direct-related hemolysin (TRH) are the well-known virulence factors in *V. parahaemolyticus*. TDH is a pore-forming and heat stable protein which remains unharmed even heating at 100°C for 10 min (Yanagihara et al., 2010). Two distinct biological characteristics of TDH are the capability of inducing hemolysis and cytotoxicity (Baba et al., 1992; Honda et al., 1992; Fabbri et al., 1998; Raimondi et al., 2000). TRH was first identified and isolated from the gastroenteritis outbreak in Maldives in 1985 (Honda et al., 1988). The isolated *V. parahaemolyticus* strains from the outbreak shown Kanagawa negative on Wagatsuma agar and did not possess the TDH genes (Nishibuchi et al., 1989). Unlike TDH thermostability, TRH is a heat labile protein at 60°C for 10 min (Honda et al., 1988). TRH shares similar biological, immunological, and physicochemical characteristics with TDH have been described (Honda and Iida, 1993). For instance, TRH induces Cl^−^ secretion in cultured human colonic epithelial cells by a similar mechanism with TDH (Takahashi et al., 2000).

Antimicrobial resistance (AMR) has now been recognized as a significant global threat issue to global public health and food security (FAO, 2016). Many frequently used antibiotics are no longer effective to control infections. Extensive use and misuse of antibiotics in agriculture, aquaculture, and livestock production are considered to be the main factor leads to the emergence and spread of AMR. Multidrug-resistant (MDR) bacterial strain is another emerging challenge when a bacterial cell becomes resistant toward multiple antibiotics. Development of MDR bacterial strains can be achieved through several mechanisms such as chromosomal DNA mutations, enzymatic inactivation, transformation as well as conjugation (Van Hoek et al., 2011). Antimicrobial residues present in the environment may also resultant in selection pressure for AMR bacteria. In aquaculture, antibiotics are used in fish farming to promote the growth of aquatic organisms and control bacterial infections. Administration of antibiotics into feed and water is a usual practice to improve production growth and for the treatment of infection diseases caused by pathogenic bacteria. Several *Vibrio* species are known to cause infections in aquatic fish and extensive use of antibiotics in the past have now led to significant increases in the occurrence of AMR *Vibrio* species (Letchumanan et al., 2015b).

Quinolones, cephalosporins, tetracycline, cefotaxime, ceftazidime, and penicillins are some commonly recommended clinical antibiotics used for the treatment of non-cholerae *Vibrio* spp. infections (Han et al., 2007; Wong et al., 2015). The use of quinolone is generally effective to against all *Vibrio* species; while the use of cephalosporin and tetracycline antibiotics was reported ineffective and associated with higher mortality in patients with vibriosis (Wong et al., 2015). In aquaculture industry, tetracyclines, erythromycin, sulfonamides, oxytetracyclines, chlortetracycline, and amoxicilin are allowed to be used in some of the ASEAN countries including Malaysia, Myanmar, and Philippines; while other antimicrobial drugs such as nitrofurans, chloramphenicol, and dimetridazole/metronidazole are banned in most countries (ASEAN, 2013; Weese et al., 2015). For controlling *Vibrio* spp., sulfonamides sold under the trade name Dimeton is an example of veterinary antibiotic commonly used in hatcheries to control infections (Shariff et al., 2000).

Short mackerel (*Rastrelliger brachysoma*) is an important commercial small pelagic fish species and have a high preference among consumers because of its affordable price and widely available throughout the year in many of the Southeast Asia countries including Malaysia, Thailand, Cambodia, Philippines, and Indonesia. Numerous prevalence studies and outbreaks of *V. parahaemolyticus* infection have been extensively reported in oysters, clams, cockles, mussels, crabs, and shrimps (Newton et al., 2014; Rodgers et al., 2014; Xu et al., 2014; Malcolm et al., 2015). In finfish species, qualitative detection of *V. parahaemolyticus* in the anchovies (*Engraulis* spp.), gray mullet (*Mugil cephalus*), red mullet (*Mullus surmuletus*), sardines (*Sardina* spp.), and Atlantic mackerel (*Scomber scombrus*) have been examined by Baffone et al. (2000) with the used of selective medium and biochemical tests. Hara-kudo et al. (2003) examined 15 horse mackerels purchased from the marketplaces in Japan, for *tdh* positive and total *V. parahaemolyticus* by using PCR and CHROMagar Vibrio (CV) agar. Reports focused on the detection and enumeration of *V. parahaemolyticus* in finfish species such as mackerel is very limited in extent. Available data on the quantitative detection of *V. parahaemolyticus* in different parts of the mackerel's body can only be found in one study reported in a Japanese-language literature (Ohno et al., 1993). Hence, the main purpose of this study was to determine the prevalence of total and pathogenic *V. parahaemolyticus* in short mackerels purchased from different wet markets, hypermarkets, and minimarkets in the state of Selangor of Malaysia by using MPN-PCR methods and to evaluate the antibiotic susceptibility profiles of *V. parahaemolyticus* isolates obtained from the short mackerels.

## Materials and methods

### Sample collection

A total of 130 short mackerel (*R. brachysoma*) samples were collected from 67 sampling date within a period of 6 months from Jan 2016 to June 2016 in four wet markets (*n* = 42), five hypermarkets (*n* = 42), and five minimarkets (*n* = 46) in Selangor, Malaysia. A maximum of two short mackerels were collected at each sampling date from the sampling location. All the samples were transported to the laboratory and processed immediately on the day of sampling.

### Sample processing and most probable number (MPN) method

Flesh (with skin), gills, and intestines excised from each short mackerel were used as the test sample. A total of 390 test samples including of 130 fleshes, 130 gills, and 130 intestines were analyzed in this study. Ten grams of flesh was transferred into 90 ml of alkaline peptone water (APW) (Merck, Darmstadt, Germany) in a sterile stomacher bag. One gram of gills and one gram of intestines, each was transferred into 9 ml of APW in two separate sterile stomacher bags. Samples were homogenized for 1 min using a Stomacher Lab-Blender 400 (Seward Medical, UK). MPN preparation was followed the US FDA Bacteriological Analytical Manual (BAM) three tubes MPN methodology with some modifications (Kaysner and DePaola, 2004). Briefly, a serial dilution was carried out up to 10^−5^ by transferring 1 ml suspension mixture into 9 ml of APW. One milliliters of each dilution sample was pipetted into three microcentrifuge tubes and tubes were incubated at 37°C for 18–24 h.

### Genomic DNA extraction

A number of tubes with growth at each dilution was subjected to genomic DNA extraction through physical cell disruption methods. Samples with growth or turbidity were pelleted by centrifugation at 13,400 × g for 3 min. The supernatant was discarded and the remained pellet was suspended in 200 μl TE buffer. The suspension mixture was heated at 100°C for 15 min in a dry bath (Labnet, USA) and immediately kept at −20°C for another 15 min. After the heat and cold-shock cell lysis, samples were centrifuged at 13,400 × g for 1 min and the supernatant was used as DNA template for multiplex PCR.

### Multiplex PCR

Multiplex PCR was performed by using 3 set of primers: (i) toxR (F: 5′-GTCTTCTGACGCAATCGTTG-3′ and R: 5′-ATACGAGTGGTTGCTGTCATG-3′) for species-specific detection of *V. parahaemolyticus* (Kim et al., 1999); (ii) tdh (F: 5′-CCACTACCACTCTCATATGC-3′ and R: 5′-GGTACTAAATGGCTGACATC-3′) for the detection of pathogenic *tdh* gene (Tada et al., 1992); and (iii) trh (F: 5′-TTGGCTTCGATATTTTCAGTATCT-3′ and R: 5′-CATAACAAACATATGCCCATTTCCG-3′) for the detection of pathogenic *trh* gene (Bej et al., 1999). All primers were synthesized by Sigma-Aldrich (USA) and PCR protocol was carried out according to the method as described by Malcolm et al. (2015). Briefly, PCR reagents (Promega, USA) were prepared by mixing 1.4 × PCR buffer, 2.5 mM of MgCl_2_, 0.2 mM of dNTPs, 0.2 μM of each primer, 2.5U of Taq polymerase, 2 μL of DNA template and top up to the final volume of 25 μL with sterilized ultrapure water. Amplification was performed in a Kyratec SuperCycler Trinity (Australia) with the following thermocycling conditions: initial denaturation at 95°C for 5 min for 1 cycle, 30 cycles consisting of denaturation at 95°C for 30 s, annealing at 60°C for 45 s, extension at 68°C for 1 min, and a final extension at 72°C for 3 min.

### Antibiotic susceptibility test

One *V. parahaemolyticus* colony from each sampling date was collected for antibiotic susceptibility test. A total of 67 *V. parahaemolyticus* colonies isolated from short mackerel on 67 sampling date were tested for antibiotic susceptibility by using disc diffusion method. Isolates were cultured in 5 mL of Mueller-Hinton broth (Merck, Darmstadt, Germany) supplemented with 3% (w/v) of NaCl (Merck, Darmstadt, Germany) and incubated at 37°C, 120 rpm for 24 h. Inoculums were swabbed with the sterile cotton swab on the entire surface of Mueller-Hinton agar (Merck, Darmstadt, Germany) supplemented with 3% (w/v) of NaCl and left to dry for 3–5 min. Antimicrobial susceptibility test discs (Oxoid, UK) were placed on the inoculated agar plate with a disc dispenser and incubated at 37°C for 24 h. A total of 17 antimicrobial susceptibility test discs impregnated with ampicillin (10 μg), ampicillin sulbactam (20 μg), amikacin (30 μg), amoxicillin/clavulanic acid (30 μg), ceftazidime (30 μg), cefotaxime (30 μg), cephalothin (30 μg), chloramphenicol (30 μg), ciprofloxacin (5 μg), doxycycline (30 μg), gentamicin (10 μg), imipenem (10 μg), levofloxacin (5 μg), meropenem (10 μg), penicillin G (10 unit), streptomycin (10 μg), and tetracycline (30 μg) were used in this study. *Escherichia coli* ATCC 25922 was used as quality control organism for this antibiotic susceptibility test. After incubation, the diameter of inhibition zone was measured in nearest whole millimeter. Antibiotic susceptibility profile of the isolate was interpreted as sensitivity, intermediate, and resistance based on the Clinical and Laboratory Standards Institute (CLSI) M45 guideline for *Vibrio* spp. (CLSI, 2010). Interpretive criteria for doxycycline, streptomycin and penicillin G not available in the M45 guidelines was referred to CLSI M100 guideline (CLSI, 2016). Multiple antibiotic resistance (MAR) index value was calculated according to Krumperman (1983) by using the formula, a/b, where “a” is the number of antibiotics to which the particular isolate was resistant and “b” is the total number of antibiotics tested.

### Statistical analysis

Statistically significant differences among the sample microbial loads and sampling locations were analyzed with analysis of variance (ANOVA) tests using Minitab statistical package version 16.2 (Minitab Inc., State College, PA). The level of significance was set at *P* ≤ 0.05. Significant differences between the microbial concentrations in the flesh, gills, and intestines of short mackerel were examined as well.

## Results

### Total *V. parahaemolyticus* in short mackerels

Prevalence and microbial loads of total *V. parahaemolyticus* in short mackerels are summarized in Table 1. DNA fragments of 368 bp in size were produced from the amplification of *V. parahaemolyticus* species-specific gene (*toxR*) indicating the presence of total *V. parahaemolyticus*. Out of 390 short mackerel tested samples, 310 (79.5%) samples were detected with the presence of total *V. parahaemolyticus* (*toxR*) and the microbial loads was between <3 to >10^5^ MPN/g. Highest prevalence of total *V. parahaemolyticus* was detected in the samples obtained from wet markets (78.6–95.2%), followed by the minimarkets (82.6–89.1%) and hypermarkets (50.0–83.3%). Highest density of total *V. parahaemolyticus* were found in short mackerel gills with mean concentration of 3.66 log MPN/g, followed by intestines with mean concentration of 2.67 log MPN/g and flesh with mean concentration of 1.74 log MPN/g. Besides, 33.9% (105/310) of the samples were detected with high levels (≥10^4^ MPN/g) of total *V. parahaemolyticus*.

**Table 1:** Prevalence and microbial loads of total *V. parahaemolyticus* in short mackerels.

**Sampling location**	**Samples**	**Number of sample**	**Number of positive sample (%)**	**Level of total (*****toxR*****)** ***V. parahaemolyticus*** **(MPN/g)**				
				**<100**	**10^2^**	**10^3^**	**10^4^**	**>10^5^**
Wet market	Flesh	42	39 (92.9%)	19	6	8	2	4
	Gills	42	40 (95.2%)	1	4	17	8	10
	Intestines	42	33 (78.6%)	1	8	6	9	9
Hypermarket	Flesh	42	23 (54.8%)	17	3	3	–	–
	Gills	42	35 (83.3%)	**–**	7	19	6	3
	Intestines	42	21 (50.0%)	–	8	10	2	1
Minimarket	Flesh	46	40 (87.0%)	20	11	5	1	3
	Gills	46	41 (89.1%)	–	2	9	12	18
	Intestines	46	38 (82.6%)	1	9	11	6	11
	Total=	390	310 (79.5%)	59	58	88	46	59

Overall, the prevalence of total *V. parahaemolyticus* in short mackerel whole-body was 89.2% (116/130). A total of 95.2% (40/42), 89.1% (41/46), and 83.3% (35/42) of the short mackerel samples obtained from the wet markets, minimarkets, and hypermarkets, respectively, were detected with the presence of total *V. parahaemolyticus*. For statistical analyses, samples collected from the hypermarkets was detected significant lower (*P* < 0.05) from the wet markets and minimarkets. The level of total *V. parahaemolyticus* in short mackerel flesh, gills, and intestines were also showed significantly different (*P* < 0.05) from each other.

### Pathogenic *V. parahaemolyticus* strains in short mackerels

Prevalence and microbial loads of pathogenic *V. parahaemolyticus* (*tdh*+ and/or *trh*+) strains in short mackerels are shown in Tables 2, 3. DNA fragments of 484 and 251 bp in size were produced from the amplification of *V. parahaemolyticus* pathogenic *trh* and *tdh* genes, respectively. A total of 33 out of 390 (8.5%) tested samples were detected positive for the *tdh* gene (*tdh*+ and *trh*−) and the microbial loads ranged from 3.6 to >10^5^ MPN/g. Strains of *V. parahaemolyticus* harbored both the *tdh* and *trh* genes (*tdh*+ and *trh*+) were detected in 4 out of 390 (1.0%) tested samples and the density of *tdh*+ and *trh*+ *V. parahaemolyticus* strains found in the samples ranged from 300 to 740 MPN/g. *V. parahaemolyticus* strain carrying only the *trh* gene (*trh*+ and *tdh*-) was not detected in this study.

**Table 2:** Prevalence and microbial loads of pathogenic *tdh*+ and *trh*− *V. parahaemolyticus* strains in short mackerels.

**Sampling location**	**Samples**	**Number of sample**	**Number of positive sample (%)**	**Level of (*tdh*+ and *trh*−) *V. parahaemolyticus* (MPN/g)**				
				**<100**	**10^2^**	**10^3^**	**10^4^**	**>10^5^**
Wet market	Flesh	42	2 (4.8%)	2	–	–	–	–
	Gills	42	4 (9.5%)	1	1	1	1	
	Intestines	42	3 (7.1%)	–	1	–	–	2
Hypermarket	Flesh	42	3 (7.1%)	2	1	–	–	–
	Gills	42	4 (9.5%)	–	1	2	–	1
	Intestines	42	3 (7.1%)	–	2	–	–	1
Minimarket	Flesh	46	5 (10.9%)	2	–	–	1	2
	Gills	46	5 (10.9%)	–	1	1	1	2
	Intestines	46	4 (8.7%)	–	1	1	–	2
	Total=	390	33 (8.5%)	7	8	5	3	10

**Table 3:** Prevalence and microbial loads of pathogenic *tdh*+ and *trh*+ *V. parahaemolyticus* strains in short mackerels.

**Sampling location**	**Samples**	**Number of sample**	**Number of positive sample (%)**	**Level of (*****tdh***+ **and** ***trh***+**)** ***V. parahaemolyticus*** **(MPN/g)**				
				**<100**	**10^2^**	**10^3^**	**10^4^**	**>10^5^**
Wet market	Flesh	42	0 (0%)	–	–	–	–	–
	Gills	42	0 (0%)	–	–	–	–	–
	Intestines	42	2 (4.8%)	–	2	–	–	–
Hypermarket	Flesh	42	0 (0%)	–	–	–	–	–
	Gills	42	1 (2.4%)	–	1	–	–	–
	Intestines	42	1 (2.4%)	–	1	–	–	–
Minimarket	Flesh	46	0 (0%)	–	–	–	–	–
	Gills	46	0 (0%)	–	–	–	–	–
	Intestines	46	0 (0%)	–	–	–	–	–
	Total=	390	4 (1.0%)	–	4	–	–	–

Overall, the prevalence of pathogenic *V. parahaemolyticus* strain (*tdh*+ and/or *trh*+) in short mackerel whole-body was 16.2% (21/130). The presence of pathogenic *V. parahaemolyticus* strains can be found in either combination or alone in each part of short mackerel flesh, gills, and intestines. For statistical analyses, the level of pathogenic (*tdh*+ and/or *trh*+) *V. parahaemolyticus* was detected non-significantly different (*P* > 0.05) in various sampling locations and tested samples.

### Antibiotics susceptibility profile of *V. parahaemolyticus* isolates

Antibiotic susceptibility profiles of *V. parahaemolyticus* isolated from short mackerel samples are shown in Table 4. The majority of isolates tested were susceptible to all of the antibiotics. Isolates tested were highly susceptible to antibiotics such as ampicillin sulbactam (100%), meropenem (100%), ceftazidime (98.5%), and imipenem (98.5%). High level of resistance was observed to penicillin G (92.5%) and ampicillin (82.1%). Of 67 isolates, 60 (89.6%) showed resistant to two or more antibiotics. MAR index value of *V. parahaemolyticus* isolates from short mackerel samples are summarized in Table 5. Most of the isolates (40.3%) were detected with MAR index value of 0.12 followed by 16.4 and 13.4% of isolates with MAR index value of 0.29 and 0.24, respectively. Two (3.0%) isolates exhibited the highest MAR index value of 0.41 which shown resistance to 7 types of antibiotics.

**Table 4:** Antibiotic susceptibility profiles of *V. parahaemolyticus* isolated from short mackerels by disc diffusion method.

**Antibiotics**	**Interpretive category result**		
	**Resistant (%)**	**Intermediate (%)**	**Sensitivity (%)**
Ampicillin (10 μg)	55 (82.09)	5 (7.46)	7 (10.45)
Ampicillin sulbactam (20 μg)	**–**	–	67 (100)
Amikacin (30 μg)	15 (22.39)	8 (11.94)	44 (65.67)
Amoxicillin/clavulanic acid (30 μg)	3 (4.48)	3 (4.48)	61 (91.04)
Ceftazidime (30 μg)	–	1 (1.49)	66 (98.50)
Cefotaxime (30 μg)	1 (1.49)	21 (31.34)	45 (67.16)
Cephalothin (30 μg)	21 (31.34)	14 (20.90)	32 (47.76)
Chloramphenicol (30 μg)	6 (8.96)	–	61 (91.04)
Ciprofloxacin (5 μg)	3 (4.48)	25 (37.31)	39 (58.21)
Doxycycline (30 μg)	2 (2.99)	2 (2.99)	63 (94.03)
Gentamicin (10 μg)	7 (10.45)	10 (14.93)	50 (74.63)
Imipenem (10 μg)	–	1 (1.49)	66 (98.50)
Levofloxacin (5 μg)	1 (1.49)	8 (11.94)	58 (86.57)
Meropenem (10 μg)	–	–	67 (100)
Penicillin G (10 μg)	62 (92.54)	–	5 (7.46)
Streptomycin (10 μg)	36 (53.73)	9 (13.43)	22 (32.84)
Tetracycline (30 μg)	6 (8.96)	5 (7.46)	56 (83.58)

**Table 5:** Multiple antibiotic resistance (MAR) index value of *V. parahaemolyticus* isolates from short mackerel samples.

**MAR index**	**Antibiotic resistance profiles^a^**	**Isolates code**	**Percentage of isolates (%)**
0.41	Amp, Ak, Amc, Kf, C, P, S	V8	1.5
	Amp, Ak, Kf, C, Cip, P, S	V11	1.5
0.35	Amp, Ak, Kf, Cn, P, S	V80, V85	3.0
	Amp, Ak, Amc, Kf, C, P	V9	1.5
0.29	Amp, Ak, Kf, P, S	V40, V87, V89	4.5
	Amp, Kf, Cn, P, S	V81, V91	3.0
	Amp, Kf, C, P, Te	V5	1.5
	Amp, Amc, C, P, S	V7	1.5
	Amp, Kf, Cip, P, S	V51	1.5
	Amp, Cn, Lev, P, S	V53	1.5
	Amp, Ak, Cn, P, S	V54	1.5
	Amp, Ak, C, P, S	V54	1.5
0.24	Amp, Kf, P, S	V46, V78, V84, V92	6.0
	Amp, Ak, Kf, P	V39, V58	3.0
	Amp, Kf, P, Te	V25	1.5
	Amp, Ak, P, S	V52	1.5
	Amp, Cn, P, S	V90	1.5
0.18	Amp, P, S	V44, V50, V86	4.5
	Amp, Kf, P	V16	1.5
	Amp, Ak, P	V26	1.5
	Ctx, Kf, P	V38	1.5
	Ak, Cip, S	V57	1.5
0.12	Amp, P	V1, V3, V10, V12, V13, V18, V27-37, V43, V45, V48, VP72-75, V77	37.3
	Do, Te	V22, V23	3.0
0.06	P	V4, V6, V14, V19, V41, V42	9.0
	Te	V20, V21	3.0

a *Amp, ampicillin; Ak, amikacin; Amc, amoxicillin/clavulanic acid; Ctx, cefotaxime; Kf, cephalothin; C, chloramphenicol; Cip, ciprofloxacin; Do, doxycycline; Cn, gentamicin; Lev, levofloxacin; P, penicillin G; S, streptomycin; Te, tetracycline*.

## Discussion

High prevalence and microbial loads of total *V. parahaemolyticus* were detected in 89.2% (116/130) of short mackerel samples. Similar results were found in the Hara-kudo et al. (2003) study where 15 out of 17 (88.2%) horse mackerel samples in Japan were detected with the presence of total *V. parahaemolyticus*. However, the incidence rate of total *V. parahaemolyticus* in short mackerels was comparatively higher than many other prevalence studies in other countries. For instance, Baffone et al. (2000) revealed 2.6% (3/114) fish samples including of anchovies, gray mullet, red mullet, sardines, Atlantic mackerel, and other species common to the Adriatic Sea were detected with the presence of *V. parahaemolyticus*. Wong et al. (1999) reported 29.3% of a total 92 fish samples imported from Indonesia were detected with total *V. parahaemolyticus*. Nakaguchi (2013) reported an average of 57.4% fish samples obtained from Asia countries including Vietnam, Indonesia, and Malaysia were found with total *V. parahaemolyticus*. Total *V. parahaemolyticus* were found more prevalently in the samples obtained from wet markets (78.6–97%) followed by minimarkets (82.6–89.1%) and hypermarkets (50.0–83.3%) in this study. Similar study findings reported by Letchumanan et al. (2015a) also found a high level of *V. parahaemolyticus* contamination in shrimp samples purchased from wet markets compared to supermarkets. The reason for these findings may due to the poor storage handling practices by the wet market retailers. For instance, seafood especially cheaper fishes such as short mackerels in the wet markets were usually displayed on a tray with little or no ice left amongst the fish. Additionally, finfish are generally sent directly from the farm to wet markets retailers under minimum temperature controlled.

Microbial loads of total *V. parahaemolyticus* in short mackerel tested samples including flesh, gills, and intestines obtained from the hypermarkets were detected significant lower (*P* < 0.05) from the wet markets and minimarkets. The study findings are in agreement with another study reported the surf clams obtained from hypermarkets were found to contain lower density of *V. parahaemolyticus* (Malcolm et al., 2015). In general, seafood is extremely perishable which needs strictly controlled storage requirements to maintain their freshness and limit the growth of harmful microorganisms. In this study, samples obtained from hypermarkets were found to contain significant lower number of total *V. parahaemolyticus* (*P* < 0.05). This might due to the well-established cold chains and cold storage facilities available in the hypermarkets. Low temperature controlled in supply chains and storage facilities available in the hypermarkets which therefore help reduce the growth of total *V. parahaemolyticus* in short mackerels. Su and Liu (2007) also reported that *V. parahaemolyticus* are cold sensitive thus preserving seafood on low temperature may limit or reduce their growth.

Short mackerel gills were detected with the highest total *V. parahaemolyticus* microbial loads with a mean concentration of 3.66 log MPN/g compared to intestines with a mean concentration of 2.67 log MPN/g and flesh with a mean concentration of 1.74 log MPN/g. Hairy structures of gills provided large surface areas for the bacteria adsorption is suggested one of the reasons where short mackerel gills were detected with the highest density of total *V. parahaemolyticus*. In Malaysia, there is no standard safety limit or minimum allowable level of *V. parahaemolyticus* in seafood. Safety regulations and standards for fish and fishery product established from other countries such as the United States are referred. According to the FDA microbiological safety limits for *V. parahaemolyticus* in ready-to-eat fish products must be <1 × 10^4^ per gram (FDA, 2011). In this study, 33.9% of short mackerels exceed the microbiological safety levels but fish evisceration, washing, rinsing, and cooking process done by food handlers during food preparation is suggested capable of achieving certain reductions of *V. parahaemolyticus*. For examples, Watanabe et al. (1994) demonstrated the effectiveness of washing of the horse mackerel eviscerated cavity with clean water resulted in 1.99 log reduction of *V. parahaemolyticus*. Ye et al. (2012) reported mild heat treatment at 50°C for 20 min was capable of reducing the number of *V. parahaemolyticus* to below detection limit (<3 MPN/g).

Most of the *V. parahaemolyticus* clinical isolates exhibit Kanagawa positive or negative possessing the hemolysin *tdh* and/or *trh* genes. Only small percentage of *V. parahaemolyticus* isolates from food and environmental samples carrying *tdh* and/or *trh* genes. This statement was in agreement with our findings as *V. parahaemolyticus* strains with *tdh* gene and strains with both *tdh* and *trh* genes were detected in low level, 8.5 and 1.0%, respectively, in short mackerels. No *trh*+ *V. parahaemolyticus* strains was found in all samples can be associated with the warmer climate in Malaysia as Rodriguez-Castro et al. (2010) reported that *trh*+ strains more prevailing in the coldest water and *tdh*+ *V. parahaemolyticus* tends to disseminate in the warmer water. Even in clinical *V. parahaemolyticus* strains only minority of the isolates carried the *trh* genes. Suthienkul et al. (1995) reported merely 10 out of 489 (2%) *V. parahaemolyticus* isolates isolated from patients with acute gastroenteritis possessed the *trh* genes and 27 out of 489 (6%) isolates harbored both the *trh* and *tdh* genes. Bhoopong et al. (2007) revealed only 0.5% (3/629) of the clinical *V. parahaemolyticus* isolates from the 63 patients in Thailand carried the *trh* gene alone, compared to 87.4% (550/629) and 7% (44/629) of the isolates possessed the *tdh* gene and both genes, respectively. Chen et al. (2016) reported 93% and 1% of the 501 clinical *V. parahaemolyticus* isolates from southeastern China were carried *tdh* gene and *trh* gene, respectively. However, distributions of *tdh*+ and/or *trh*+ strains may vary depend on the geographical region, sample source, and detection method (Raghunath, 2015).

*V. parahaemolyticus* isolates obtained from short mackerels displayed a high level of resistance to penicillin and ampicillin. This finding was consistent with the results reported by Letchumanan et al. (2015a) where 82% of the isolates from shrimp samples were resistant to ampicillin. Besides, Elexson et al. (2014) reported all of the *V. parahaemolyticus* isolates from cultured seafood products were resistant to both penicillin and ampicillin. Based on these findings, resistant to penicillin and ampicillin could be probably due to extensively used of antibiotics in aquaculture and the impact of antimicrobial residues in aquatic systems. It is also likely due to the complexity of the Gram-negative bacteria outer membrane which inhibits antibiotic compounds to pass through the outer membrane (IFT, 2006; Blair et al., 2014). Penicillin and ampicillin are therefore ineffective for the treatment of *V. parahaemolyticus* infections. Nevertheless, the majority of the isolates were susceptible to most antibiotics tested in this study. Susceptibility profiles of *V. parahaemolyticus* isolates to antibiotic classes such as tetracycline, phenicols, quinolone, and cephalosporin were comparable with many other studies reported in several countries and sample sources (Lesmana et al., 2001; Han et al., 2007; Yano et al., 2011; Ottaviani et al., 2013). In this study, 37.31% of isolates with MAR value more than 0.2 indicating samples originated from a high risk source of contamination where several antibiotics are used (Krumperman, 1983). Widespread usage of antibiotics in clinical, agriculture, aquaculture, and livestock production can result in antimicrobial residues present in the environment and dispersion of antimicrobial residues that reach the marine environment could lead to selective pressure on the marine bacteria and the emergence of MDR bacterial strains in marine life.

Misuse and overuse of antibiotics are recognized as two of the major factors contribute to the development of resistance genes in bacteria and widespread dissemination of MDR bacterial strains. An alternative to antibiotics is urgently needed in order to overcome the continuous emergence of MDR bacterial strains in the environment (Tan et al., 2016). Research on alternative approaches such as the use of bacteriophage and probiotics has become possible solution to replace or reduce the use of antibiotics. Bacteriophages are bacteria viruses with the ability to attack and destroy bacteria cells. Jun et al. (2014) demonstrated bacteriophage-based therapies displays effectual protection against multiple antibiotics resistant *V. parahaemolyticus* strains infection in mice. Lomelí-Ortega and Martínez-Díaz (2014) reported two isolated lytic bacteriophages can be used to control vibriosis in whiteleg shrimp larvae (*Litopenaeus vannamei*). Probiotics are organisms or substances that bring beneficial effect to the host (Hai, 2015). Tan et al. (2016) proposed the feed supplemented with the genus of *Streptomyces* bacteria as probiotics could protect aquaculture livestock from pathogens and enhance the growth performance of the aquatic cultured organisms. Augustine et al. (2016) demonstrated *Streptomyces rubrolavendulae* M56 biogranules showed a competitive exclusion effect on *V. alginolyticus, V. parahaemolyticus, V. fluvialis*, and *V. harveyi* can be used as a promising alternative to the use of antibiotics in the prawn larval production systems. Although the application of bacteriophages and probiotics offer promising alternative to antibiotics, efforts for continuous monitoring of *V. parahaemolyticus* antibiotic resistance patterns and risk assessment on the use of antibiotics in therapeutic and non-therapeutic purposes is still needed to overcome the development of MDR bacterial strains.

## Conclusion

*V. parahaemolyticus* not only accumulates in shellfish and crustaceans but also can be found in the short mackerels at high concentrations. *V. parahaemolyticus* multiply more rapidly and reach the highest concentrations during warmer months. In Malaysia, warm temperatures remain fairly constant all year round thus it is expected high initial concentrations of *V. parahaemolyticus* can be found in the seafood. Preserving seafood at low temperature conditions can limit and control the growth of *V. parahaemolyticus*. Unbroken cold chain and cold storage facilities available in the hypermarkets maintained seafood products freshness as well as minimized the growth of foodborne pathogen such as *V. parahaemolyticus*. The low detection rate of pathogenic *V. parahaemolyticus* strains in the short mackerels may still at risk for food poisoning if storage conditions and preparation procedures are not receiving proper attention.

## Author contributions

CT is the corresponding author for this work. TM, CK, TT, WC, YL, JP, OR, MN, and NY provided assistance and guidance in throughout the research. YR, YN, MN, and SR are the mentor in the research study and assist in manuscript checking.

### Conflict of interest statement

The authors declare that the research was conducted in the absence of any commercial or financial relationships that could be construed as a potential conflict of interest.
